# Erythromycin induces lipid redistribution and toxicological effect of the marine diatom *Phaeodactylum tricornutum*

**DOI:** 10.3389/fpls.2026.1794615

**Published:** 2026-04-20

**Authors:** Shou-Fu Zhang, Jiang-Hao Mao, Jing-Ling Deng, Chun-Hai Zhao, Zhi-Peng Wang, Jing Jiang

**Affiliations:** 1 School of Environmental Science and Engineering, Suzhou University of Science and Technology, Suzhou, Jiangsu, China; 2 School of Marine Science and Engineering, Qingdao Agricultural University, Qingdao, Shandong, China; 3 Binzhou Polytechnic, Binzhou, Shandong, China

**Keywords:** erythromycin, lipidmetabolism, lipidomics, *Phaeodactylum tricornutum*, toxicological effect

## Abstract

**Introduction:**

Erythromycin poses significant ecological risks to marine ecosystem due to its persistence and lipophilic properties. However, the response mechanism of marine microalgae to erythromycin remains inadequately understood.

**Methods:**

*Phaeodactylum tricornutum* was exposed to 0–40 mg/L erythromycin; growth, photosynthetic pigments, and oxidative stress markers were measured. Nile red staining, lipidomics, and transcriptomics were used to analyze lipid remodeling and metabolic pathway changes.

**Results:**

High-dose erythromycin exposure concentration-dependently inhibited algal growth, disrupted photosynthetic pigments, and induced oxidative stress. Notably, erythromycin triggered a pronounced lipid redistribution, characterized by altered glycerolipid and glycerophospholipid profiles, increased lipid accumulation, and enhanced unsaturated fatty acid profile. Transcriptomic analyses confirmed that ERY-induced lipidome remodeling affected critical pathways to provide precursors and reducing power degradation for fatty acid synthesis, and the pathway of fatty acid degradation.

**Conclusion:**

These findings elucidate the intrinsic mechanistic link between antibiotic-induced lipid dysregulation and the physiological resilience of diatoms, providing novel insights into the molecular toxicology of antibiotics in primary producers.

## 1 Introduction

Marine ecosystems are increasingly threatened by emerging contaminants, among which antibiotics have garnered significant attention due to their persistent presence and potential ecological risks. Antibiotic consumption in the world in 2030 is expected to be increased to 200% higher than the level in 2015, which reflects the extensive demand for antibiotics (Hom-Diaz et al., 2022; Klein et al., 2018; Minh et al., 2009; Shen et al., 2024). Erythromycin (ERY), a widely used macrolide antibiotic in medicine and modern agriculture, has been frequently detected in freshwater and marine systems, where its highest concentration is 75.5 and 1.9 μg/L, respectively (Schafhauser et al., 2018−Arpin-Pont et al., 2016). It is more persistent under simulated solar irradiation than other macrolides (Batchu et al., 2014). However, exposure to ERY can induce unintended adverse effects on various organisms, including bacteria, microalgae, and invertebrates (Ji et al., 2012; Schafhauser et al., 2018). Owing to its extensive harmful effects and resistance to environmental degradation, ERY tends to bioaccumulate in marine food webs, thus being identified as one of the key pollutants requiring prioritized management in aquatic environments (Mo et al., 2023b).

ERY specifically binds to the 50S subunit of ribosomes to inhibit protein synthesis and is supposed to mainly target gram-negative and positive bacteria (Nie et al., 2013). Sendra et al. compared the inhibitory effects of different antibiotics on marine microalgae, including green algae, cyanobacteria, and diatoms and then concluded that ERY was the most toxic antibiotic to phytoplankton (Sendra et al., 2018). Most previous studies of ERY focused on cyanobacteria and green algae, with cyanobacteria showing greater sensitivity to toxicity than green algae. Generally, the 6-day EC_50_ for cyanobacteria, e.g., *Mycrocystis flos-aquae*, *Mycrocystis aeruginosa* and *Synechocystis* sp., is ranged from 23 to 430 μg/L, while the 4-d EC_50_ for *Dunaliella tertiolecta* and *Chlorella vulgaris* is 5.75 mg/L and higher than 26.33 mg/L, respectively (MaChado and Soares, 2019; Guo et al., 2021; Ma et al., 2021; Zhang et al., 2021; Le et al., 2023; Ainas et al., 2025; Touzout et al., 2025). In contrast, relatively limited information is available regarding the risk assessment and environmental monitoring of ERY for marine diatoms. *Thalassiosira weissflogii* is demonstrated to have a hormesis effect under exposure to 1 μg/L ERY. When the concentration rises to 0.75 and 2.5 mg/L, its growth and photosynthesis are inhibited with increased reactive oxygen species (ROS) formation, and multiple fundamental metabolic signaling pathways are disrupted (Mo et al., 2023a). For *Cylindrotheca closterium*, *Chaetoceros gracilis*, and *P. tricornutum*, the 3-day EC_50_ of these marine diatoms are below 1 mg/L, and high concentrations of ERY can inhibit growth, reduce Fv/Fm ratios, and elevate ROS formation in *P. tricornutum* (Sendra et al., 2018). All these studies on microalgae primarily probe into classic physiological and biochemical endpoints.

Ecotoxicological effects of ERY on microalgae highlight the differential sensitivity of algal taxa. The lipophilic nature of ERY may enhance its interaction with eukaryotic organelles related to lipid metabolism. Microalgae growth inhibition induced by ERY is also reviewed by some analyses, accompanied by pronounced shifts in lipid accumulation and composition. *Tetradesmus obliquus* displays unique vulnerability to ERY -mediated membrane remodeling. It exhibits elevated polyunsaturated fatty acid (PUFA) content, which potentially reflects adaptive responses to maintain membrane fluidity under stress (Du et al., 2020). ERY is also capable of significantly boosting the activity of acetyl-CoA carboxylase and lipid storage in *T. obliquus* (Wang et al., 2021). Besides, the accumulation of steroid metabolites was significantly enhanced under 0.001 mg/L ERY treatment, potentially promoting the growth of *T. weissflogii* at this concentration (Wu et al., 2024). Thus, a critical gap remains concerning the mechanistic basis of ERY-induced lipid metabolic reprogramming in microalgae despite its established role as a central adaptive strategy for survival and resilience under environmental perturbation.

Diatoms account for approximately 40% of global marine primary productivity and serve as food for zooplankton (Armbrust et al., 2004). *P. tricornutum*, a model diatom species, is extensively studied for its rapid growth, well-characterized genome, powerful photosynthetic efficiency, and significant lipid accumulation (Flori et al., 2016; Zhang et al., 2024). The growth and metabolic status, particularly the dynamics of lipid metabolism, are utilized to assess the impacts on marine microalgae from various emerging pollutants and environmental factors, including sulfamethoxazole, ERY, non-dioxin-like PCB-153, surgical face masks, fluoxetine, CeO_2_ NPs, nutrient starvation, temperature, and pH (Feijão et al., 2020; Xiong et al., 2020; Sendra et al., 2022; Xia et al., 2024; Zhang et al., 2024; Duarte et al., 2025). All these stresses lead to the accumulation of lipid droplets and substantial remodeling of glycerolipids (GL) in *P. tricornutum*. Among them, the nitrogen starvation-induced lipid accumulation and fatty acid changes have been thoroughly investigated for its economic benefits in the accumulation of PUFA (Jaussaud et al., 2020). Although ERY is known to significantly perturb lipid homeostasis in *P. tricornutum*, it remains unclear whether such lipid remodeling represents a passive consequence of cellular damage or an active, regulated adaptive response to intracellular antibiotic stress. Moreover, the potential synergistic or antagonistic interactions between ERY exposure and nitrogen limitation, a prevalent environmental stressor in marine systems, on diatom lipid metabolism remain entirely uncharacterized.

Therefore, this study employs the model *P. tricornutum* to address this critical gap. We hypothesize that high-dose ERY exposure triggers a systemic lipidome remodeling in *P. tricornutum*, and under nitrogen limitation, metabolic reconfiguration functions as an adaptive response while mitigating growth inhibition. The EC_50_ for different physiological parameters of *P. tricornutum* exposed to ERY ranged from 1.31 to 100 mg/L (Xiong et al., 2020). Thus, to prevent potential hormesis effects, we exposed the microalgae to milligram-level ERY stress to investigate its impact on lipid accumulation. The novelty of this work lies in its lipid-centric, multi-omics framework, which moves beyond conventional descriptive toxicity assessments to establish a mechanistic link between antibiotic-induced lipidome reprogramming and algal stress tolerance. Integrating physiological phenotyping, targeted lipidomics, and transcriptomics profiling across normal and nitrogen-limited conditions, this study delivers a systems-level understanding of how marine diatoms dynamically rewire lipid metabolism to cope with antibiotic stress, thereby advancing mechanistic insight into the ecological consequences of antibiotic pollution in marine.

## 2 Materials and methods

### 2.1 Microalga and pre-culture

The *P. tricornutum* X1 was isolated from the marine area of Qingdao and kept in a liquid nitrogen container before experiments. The microalga was incubated in f/2 marine medium (Guillard and Ryther, 1962) until the cell density of 1.5 × 10^7^. Subsequently, the obtained cells were transferred to 500 mL flasks containing 300 mL of f/2 marine medium. With an initial density of 1.0 × 10^5^ cells/mL, the cells were cultivated at 25 ± 1 °C in an artificial incubator with a light intensity of 3000 lx under a 16L:8D photoperiod. Since nitrogen is typically a limiting factor in marine environments, the algal strain was also cultivated and evaluated under nitrogen-depleted conditions. The nitrogen-deficient medium was the f/2 marine medium without NaNO_3_. To ensure the algae in suspension and facilitate CO_2_ release, the flasks were manually shaken 4 to 8 times at regular intervals daily. Algal cells in the logarithmic growth phase were taken for subsequent research.

### 2.2 ERY exposure experiments

ERY, (CAS 114-07-8, HPLC ≥ 98%) was from Solarbio, China, and other chemicals (analytical grade) were the products of Sinopharm Chemical Reagent Co., Ltd., Shanghai, China. An ERY stock solution at a concentration of 800 mg/L was diluted to 0, 5, 10, 20, 30, and 40 mg/L for exposure experiments after being freshly filtered using a 0.22 μm membrane. Culture lasted for 0, 2, 4, 6, 8, and 10 days, after which the cells were harvested for the measurements of physiological and biochemical indices. All the treatments were conducted in triplicate. ERY in culture sample was detected by HPLC-MS/MS (Waters Alliance E2695, Thermo Fisher Scientific, USA). Samples were seperated with a SunFire C18 column (50 × 2.1 mm, 2.5 μm) by following the procedure described by Wang et al (Wang et al., 2021). The ERY residue rate (R) was calculated using the equation:


$$R=C_{t}/C_{0}$$

where *C_t_* represents the ERY concentration detected in culture medium at different time, and *C_0_* is the initial concentration.

### 2.3 *P. tricornutum* growth and photosynthetic pigments content

The optical density OD_680_ of *P. tricornutum* was detected by spectrophotometry every 48 h during ERY exposure. A correlation (R^2^ > 0.99) was found between the cell density and the optical density (OD_680_) of *P. tricornutum* culture. Thus, we used OD_680_ to represent the growth of *P. tricornutum*. The growth inhibition rate of the cells in the presence of ERY was calculated as follows (Wan et al., 2015):


$$IR=(1-\frac{A}{A0})\times 100\%$$

where *A* and *A_0_* are the absorbance of the ERY treatment and the control groups, respectively, and *IR* is the inhibition ratio.

A gravimetric method was employed for biomass determination (Du et al., 2020). The algae culture was filtered through a 0.45 μm membrane and dried at 80 °C until constant mass. The equation to calculate the biomass concentration *M* (g/L) is shown below:


$$M=\;\frac{W_{2}-W_{1}}{V}$$

where *W_1_* and *W_2_* are the weights of the filter membrane before and after filtration, respectively, and *V* is the volume of the algae culture.

Photosynthetic pigments in collected cells from 10 mL algae culture were extracted overnight using 90% acetone at 4 °C. Afterward, centrifugation was performed at 5000 × *g* for 10 min at 4 °C, followed by absorbance measurement on the supernatant on a spectrophotometer. The content of chlorophyll a (Chl-a), chlorophyll c (Chl-c) and carotenoids (Car) was determined as below (Ritchie, 2006):


$$\text{Chl}–\text{a}\;(\text{μg}/\text{mL})=11.47\times A_{664}–0.40\times A_{630}$$

$$\text{Chl}–\text{c}\;(\text{μg}/\text{mL})=–3.73\times A_{664}+24.36\times A_{630}$$

$$\text{Car}\;(\text{μg}/\text{mL})=4.7\times A_{440}–1.38\times A_{662}–5.48\times A_{644}$$

where *A_664_*, *A_630_*, *A_440_*, *A_662_*, and *A_644_* are the absorbance at wavelengths of 664, 630, 440, 662, and 644 nm, respectively.

### 2.4 Measurement of intracellular ROS and antioxidative capacity

Kits for detecting ROS, total superoxide dismutase (T-SOD), catalase (CAT), malondialdehyde (MDA), and total soluble protein (TP) were all obtained from Nanjing Jiancheng Bioengineering Institute (China). Following the manufacturer’s instruction, determination of the ROS level in algae cells was achieved utilizing fluorescence probe 2’,7’-dichlorodihydrofluorescein diacetate (DCFH-DA). Soluble protein was extracted from the collected algae cells by ultra-sonication (180 W of output power) for 5 min in 0.05 M phosphate buffer (pH 7.4) and then centrifuged at 5000 rpm in 4 °C for 10 min. The total protein content, MDA concentration, and enzyme activities were determined using the supernatant. Total protein content was determined by the Bradford method (Bradford, 1976). The SOD activity was evaluated by the method of Giannopolitis and Ries (1977). One unit of its activity was defined as the amount of the enzyme required to cause a 50% inhibition rate of the reduction of water-soluble tetrazolium-1 (WST-1) at 450 nm. As for CAT, the method of Goth was adopted for activity measurement (Goth, 1991), and one unit of its activity was defined as the amount needed to degrade 1 µmol H_2_O_2_ per second per milligram of protein. The MDA concentration was measured using the thiobarbituric acid method (Duarte et al., 2025). All data were obtained from triplicates and analyzed by OriginPro software. P value < 0.05 indicates a statistically significant difference.

### 2.5 Lipid extraction and lipidomics identification

Accumulation of lipid droplets in *P. tricornutum* was monitored by Nile red (Sigma Aldrich) staining (λ_ex_ 532 nm, λ_em_ 565 nm) (Cooksey et al., 1987). The lipids were extracted by the improved chloroform-methanol method (Bligh and Dyer, 1959) and determined by gravimetric analysis. Specifically, 200 mL of algae culture was centrifuged at 5000 × *g* for 10 min. 10 mL 5 M HCl was added to the cells collected, which were then placed in an 80 °C oven for 4 h. After the acid-hydrolyzed algal liquid was cooled to room temperature, a mixture comprising chloroform/methanol (2:1, v/v, 8 mL in total) was added. The next step was 5 min of vortexed, and subsequently, 2 mL of 0.9% NaCl was added and mixed thoroughly. The mixture was allowed to stand still at room temperature for 4 h and then centrifuged at 5000 × *g* for 5 min, and the organic phase was carefully collected. The sample was concentrated to a constant weight by using a rotary vacuum evaporator and an 80 °C oven. The lipid content ω (%) was calculated as follows:


$$\text{ω}=\frac{W_{t}}{M_{t}}\times 100\%$$

where *W_t_* and *M_t_* represent the lipid weight (g/L) and the biomass concentration (g/L) after culture, respectively.

The fatty acids were trans-esterified into fatty acid methyl esters (FAMEs) (Du et al., 2020). FAMEs in the hexane phrase were analyzed by gas chromatography (TRACE 1310, Thermo Fisher Scientific, USA) coupled with flame ionization detection, with an HP-5 fused-silica capillary column (30 m × 320 μm × 0.25 μm, Agilent, USA) used. The oven temperature was set at 150 °C, then raised to 210 °C and maintained for 20 min, while the injector temperature was set at 210 °C. N_2_ was selected as the carrier gas (45 mL/min). The flow rates of H_2_ and air were 40 and 450 mL/min, respectively. The composition of fatty acids was made clear by comparing their retention time with that of standards, and chromatograms were quantified by the peak surface method. Three biological replicates were ensured in extraction and quantification.

Lipid metabolites such as glycerolipids (GL) and glycerophospholipids (GP) were then analyzed and quantified by HPLC-MS/MS (Waters Alliance E2695, Thermo Fisher Scientific, USA) with appropriate standard lipids, as described by Jaussaud (Jaussaud et al., 2020). Chromatographic separation was performed on a diol column (150 × 3 mm, 5 μm, Macherey-Nagel, Germany). Triglyceride (TG) and sulfoquinovosyl diacylglycerol (SQDG) analysis were mainly made in the negative ion mode. Phosphatidylcholine (PC), phosphatidylethanolamine (PE), phosphatidylinositol (PI), phosphatidylglycerol (PG), monogalactosyl diacylglycerol (MGDG) and digalactosyl diacylglycerol (DGDG) were mainly measured in the positive ion mode. The mass spectrometry data were analyzed using lipidsearch v.4.1 software to identify and quantify the lipid molecules detected in the ERY treated cells and control cells. Lipid classification and functional annotation was based on the KEGG pathway database. Moreover, the overall differences in lipid species between ERY treated cells and control cells were determined by principal component analysis (PCA) and partial least-squares discriminant analysis (PLS-DA). The fold change analysis was conducted, and *p*-value was calculated by the *t*-test.

### 2.6 Transcriptomic analysis

Algae from the control and the 10 mg/L ERY treatment groups were collected on day 6 for transcriptomic analysis. At this time point, the cells are in the exponential phase with highly active lipid synthesis. The biomass of each sample was no less than 5 × 10^6^ cells/mL. Total RNA of *P. tricornutum* was extracted using the Trizol method. All 6 cDNA libraries were prepared and sequenced using an Illumina NovaSeq platform (Novogene, China). The criteria to identify differentially expressed genes (DEGs) were *p* value < 0.05 and |log_2_FC| > 1. In the KEGG pathway enrichment analysis of identified DEGs, a *p* value < 0.05 was regarded as statistically significant.

## 3 Results

### 3.1 ERY exposure altered growth and lipid accumulation of *P. tricornutum*

**Figure 1:**
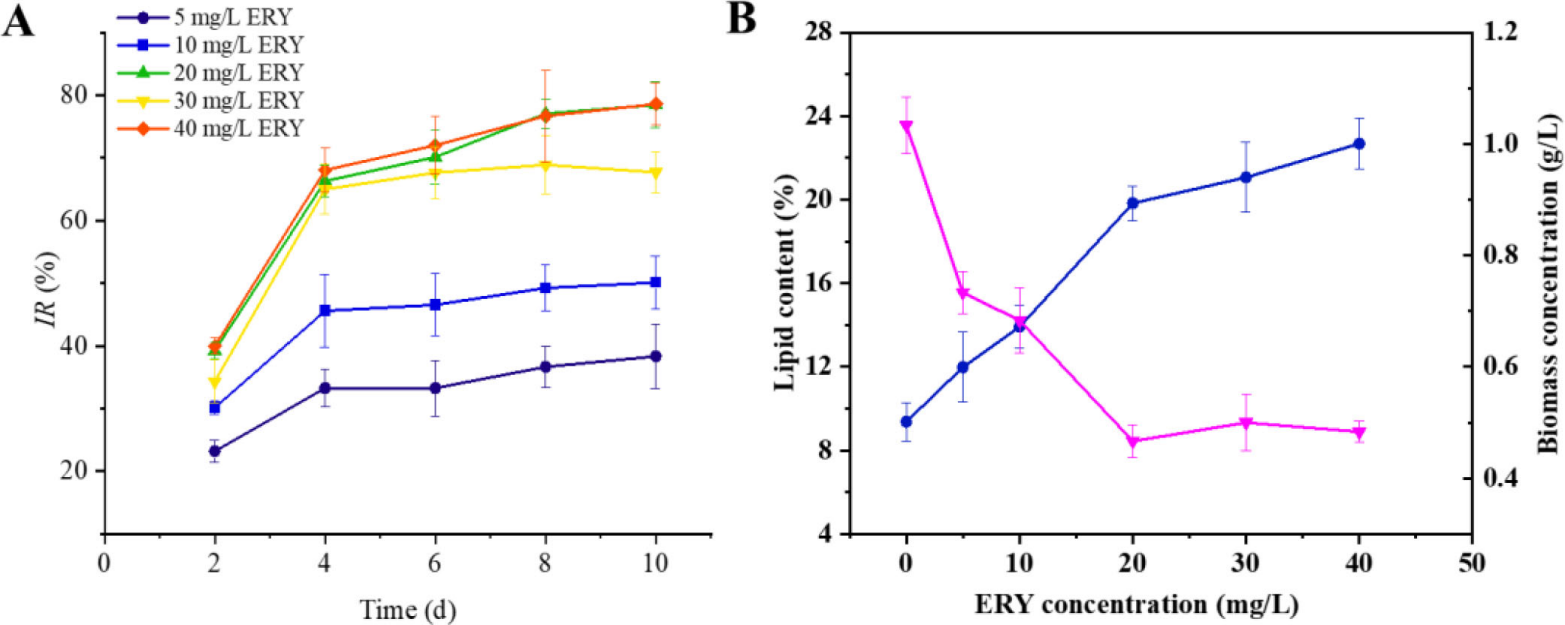
**(A)** Effects of the exposure to different concentrations of ERY on growth inhibition of *P. tricornutum*. **(B)** Effects of the exposure to different concentrations of ERY on lipid content (%) and biomass concentration (g/L) of *P. tricornutum* at the end of exposure for 10 d. The dot indicates lipid content, while the triangle denotes biomass concentration. Data are presented as mean ± SD (n = 3). Error bars indicate standard deviations°.

During the entire exposure period of 10 days, ERY at concentrations of 5–40 mg/L significantly inhibited the growth of *P. tricornutum*, with typical concentration-response curves shown (**Figure 1A**). In the case of 10 mg/L ERY, the growth inhibition rate of *P. tricornutum* was 30.14%, 45.58%, 46.54%, 49.24%, and 50.10% on days 2, 4, 6, 8, and 10, respectively. Further increasing the ERY concentration made the growth of algal cells significantly slow down and almost come to a standstill. At the end of 10 days of exposure to 40 mg/L, an inhibitory rate as high as 78.59% was observed. The distinct inhibitory effect on the growth of *P. tricornutum* was also evidenced by microscopic imaging, as shown in **Supplementary Figure S1**, where the cells in the control group are well dispersed and exhibit high viability, while those exposed to 40 mg/L ERY experience increased aggregation.

Sustained exposure to a high concentration of ERY significantly promoted lipid accumulation in *P. tricornutum* (**Figure 1B**). Specifically, the lipid content and the biomass concentration in the control group were 9.37% and 1.03 g/L at the end of 10 days of culture. As the concentration of ERY increased, the lipid content exhibited a continuous upward trend, while the biomass concentration followed a continuous downward trend. At the same time, the biomass concentration ranged from 0.73 to 0.48 g/L, which agreed with the cell density monitoring results. These data suggest that the cells may adopt lipid accumulation as a strategy to cope with the ERY stress.

### 3.2 ERY exposure altered antioxidant system of *P. tricornutum*

**Figure 2:**
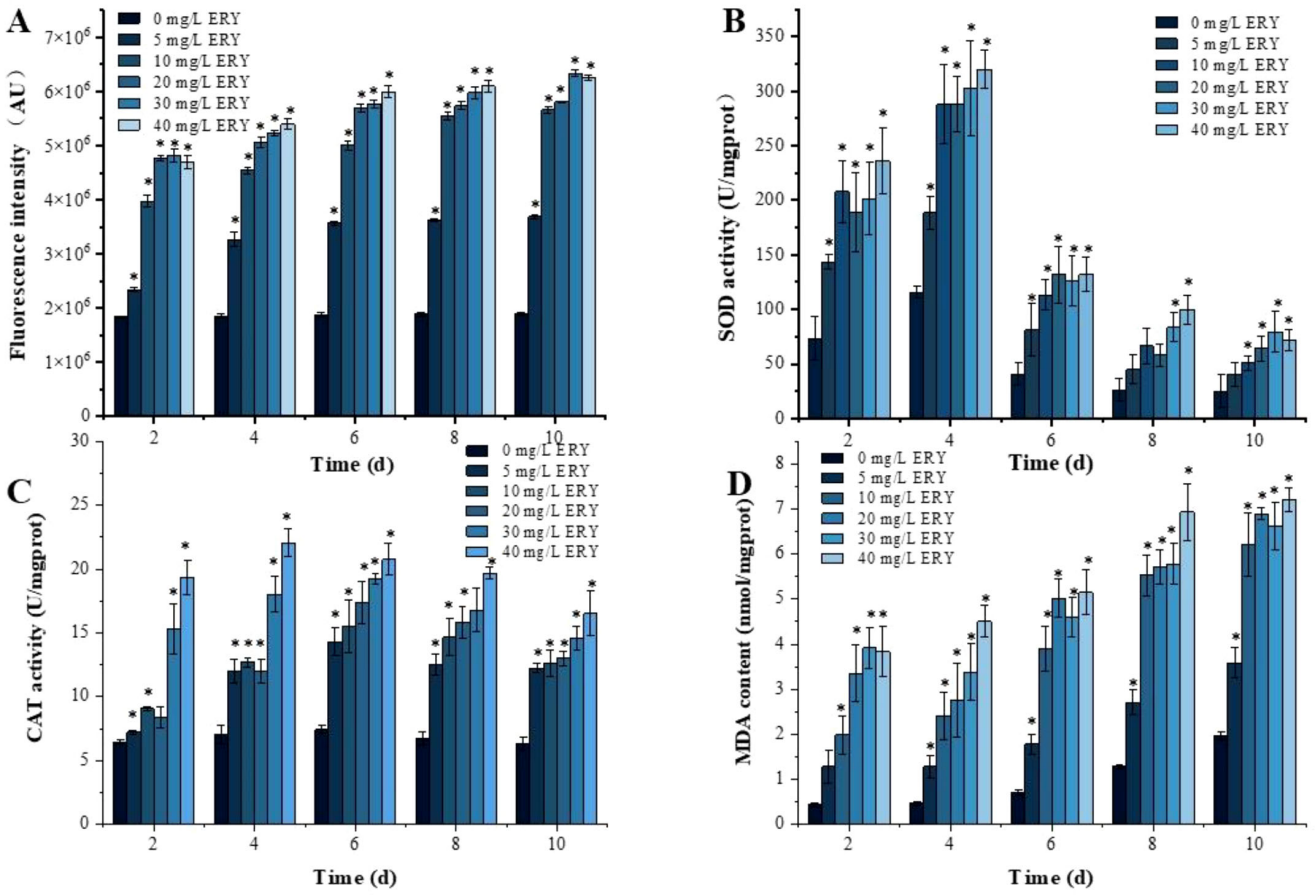
Effects of different ERY concentrations on **(A)** ROS, activities of antioxidant enzymes **(B)** SOD and **(C)** CAT, and **(D)** level of lipid peroxidation product MDA of *P. tricornutum* following a 10-day exposure. *P<0.05. Data are presented as mean ± SD (*n* = 3).

The effects of ERY on the ROS level of *P. tricornutum* are displayed in **Figure 2A**. With the prolongation of the exposure time and the increase in the ERY concentration, the accumulation of ROS gradually elevated. During the 10 days of culture, the increase in the ROS level was more pronounced when the ERY concentration was lower than 10 mg/L. Compared with the control group, the final intracellular ROS levels on the tenth day increased by 0.95, 1.99, 2.07, 2.35, and 2.30 times respectively. The accumulation of ROS in *P. tricornutum* resulted in severe oxidative damage to the cells under prolonged exposure to high concentrations of ERY.

The SOD activity and CAT activity of algal cells exhibited trends of an initial increase followed by a decrease under the influence of various concentrations of ERY (**Figures 2B, C**). The activities of the two enzymes in the ERY exposure groups were consistently higher than those in the control group, and a significant concentration-response relationship was observed. The peak activity of SOD for the cells exposed to different concentrations of ERY was observed on the fourth day, and the 5, 10, 20, 30, and 40 mg/L ERY exposure groups witnessed the elevated enzyme activity by 63%, 150%, 149%, 162%, and 177%, respectively, compared with the control group. Regarding the CAT activity, the experimental groups saw the maxima on the fourth or the sixth day. Compared with the control group, the experimental groups presented significantly increased CAT activity. In the later stages of culture, as the cells entered the senescence period, SOD activity was reduced drastically, whereas CAT activity decreased gently.

**Figure 2D** presents the alteration in the level of MDA, a biomarker for cellular oxidative damage, in algal cells over 10 days of culture. The MDA level in the ERY treated groups was markedly higher than that in the control group and rose steadily. Eventually, on the tenth day the MDA levels in the ERY treated groups were 0.82, 2.15, 2.49, 2.36, and 2.65 times that in the control group, respectively.

### 3.3 ERY exposure altered photosynthesis pigments of *P. tricornutum*

**Figure 3:**
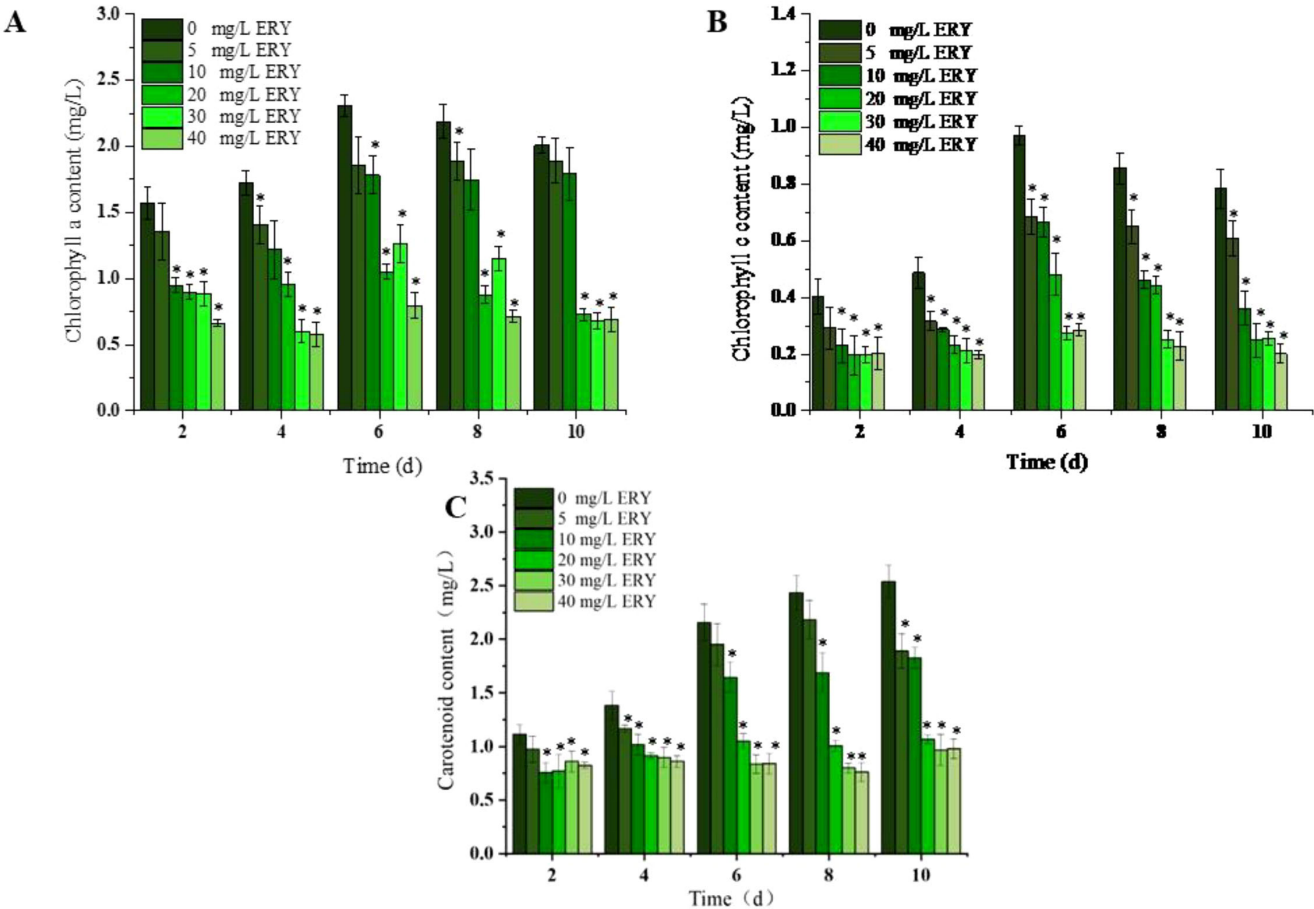
Effects of different ERY concentrations on **(A)** chlorophyll a content, **(B)** chlorophyll c content and **(C)** carotenoid content of *P. tricornutum* following 10-day exposure. *P<0.05. Data are presented as mean ± SD (n = 3).

The content of chlorophyll a and c in *P. tricornutum* increase first and then gradually decreased during the 10-day culture period, peaking on the sixth day (**Figures 3A, B**).

Their content was significantly decreased as the concentration of ERY was enhanced, which aligned with its effect on algal growth density. However, once exceeding 10 mg/L, ERY severely inhibited the synthesis of these two pigments, without conspicuous concentration-dependent variations. At the end of 10-day culture, the inhibition rates of chlorophyll a and chlorophyll c increased obviously with the introduction of 5–40 mg/L ERY.

Unlike chlorophyll a and c, carotenoid in the control group exhibited continuous increase in content throughout the culture period, which peaked on the tenth day (**Figure 3C**). With the introduction of 5–40 mg/L ERY, the carotenoid content was significantly reduced over the entire exposure period, and the reduction reached 25.51%, 28.04%, 58.00%, 61.88%, and 61.43%, respectively on the tenth day. ERY exceeding 10 mg/L resulted in consistently low carotenoid levels with minimal fluctuations over the whole culture period.

### 3.4 Alleviated growth inhibition caused by ERY under nitrogen-deficient culture

**Figure 4:**
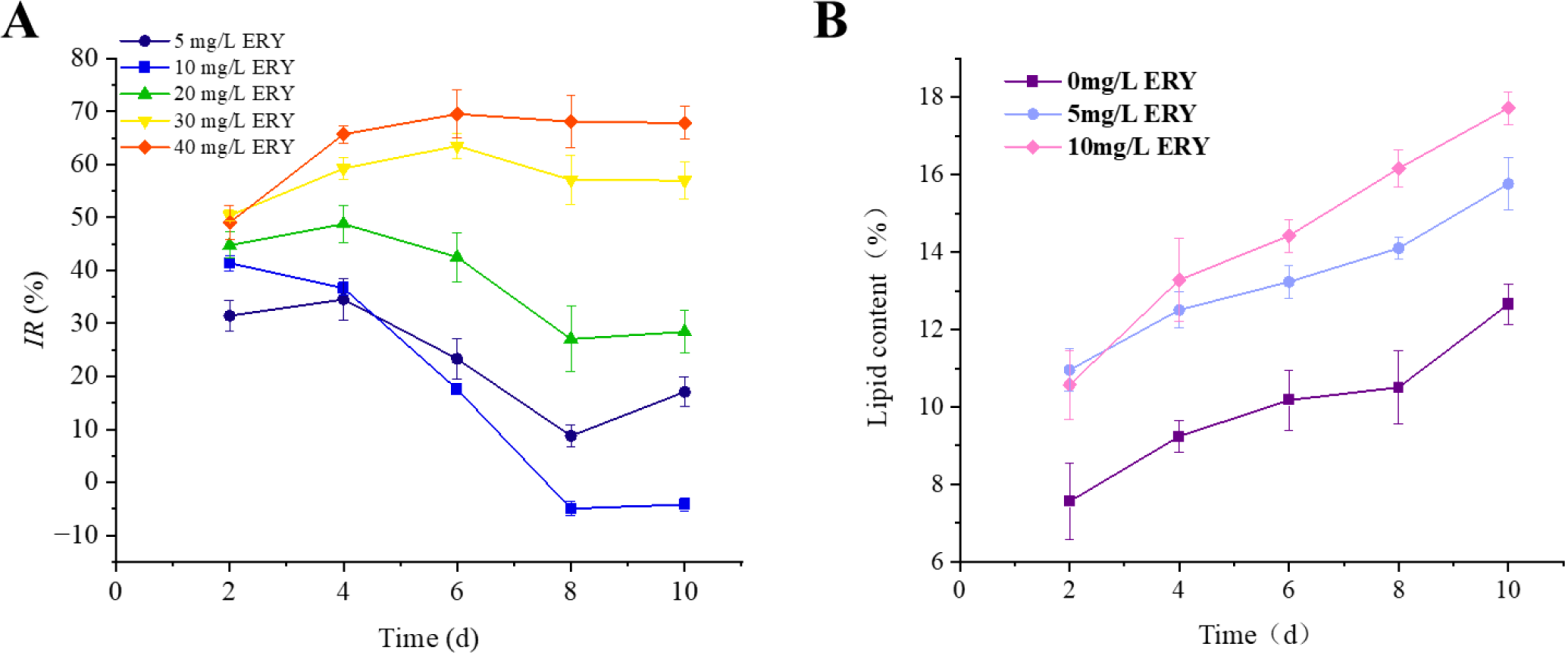
**(A)** Effects of exposure to different concentrations of ERY on growth inhibition of *P. tricornutum* in nitrogen-deficient condition. **(B)** Effects of exposure to 5 and 10 mg/L ERY on lipid content of *P. tricornutum* in nitrogen-deficient condition over 10 d of culture. Data are presented as mean ± SD (n = 3).

The inhibition rate of algal cells exposed to various concentrations of ERY decreased substantially under nitrogen-deficient culture (**Figure 4A**) compared with the normal culture (**Figure 1**). Notably, when the ERY concentration was below 20 mg/L, the inhibition rate decreased more prominently as the culture time progressed. Specifically, the growth inhibition effect of 10 mg/L ERY on *P. tricornutum* under the nitrogen-deficient condition was no longer observed after 8 days of cultivation. Despite the presence of ERY in the culture medium, lipid accumulation in *P. tricornutum* was even higher under the nitrogen-deficient condition (**Figure 4B**) than under the normal condition (**Figure 2**). To be specific, after 10 days of exposure to 5 and 10 mg/L ERY, the lipid content was 15.77% and 17.72% in the nitrogen-deficient medium, which was 11.98% and 13.91% in the normal medium. The lipid droplets in algal cells under the nitrogen-deficient condition were markedly larger than those under the normal condition (**Supplementary Figure S2**). This suggests that the lipid content may have associate with the enhanced tolerance to ERY. However, the residue rate of ERY at the end of cultivation in the two groups showed no obvious differences, which indicating that removal contribute slightly to alleviated growth inhibition (**Supplementary Figure S3**).

### 3.5 Redistribution of fatty acid composition and lipid kinds

**Figure 5:**
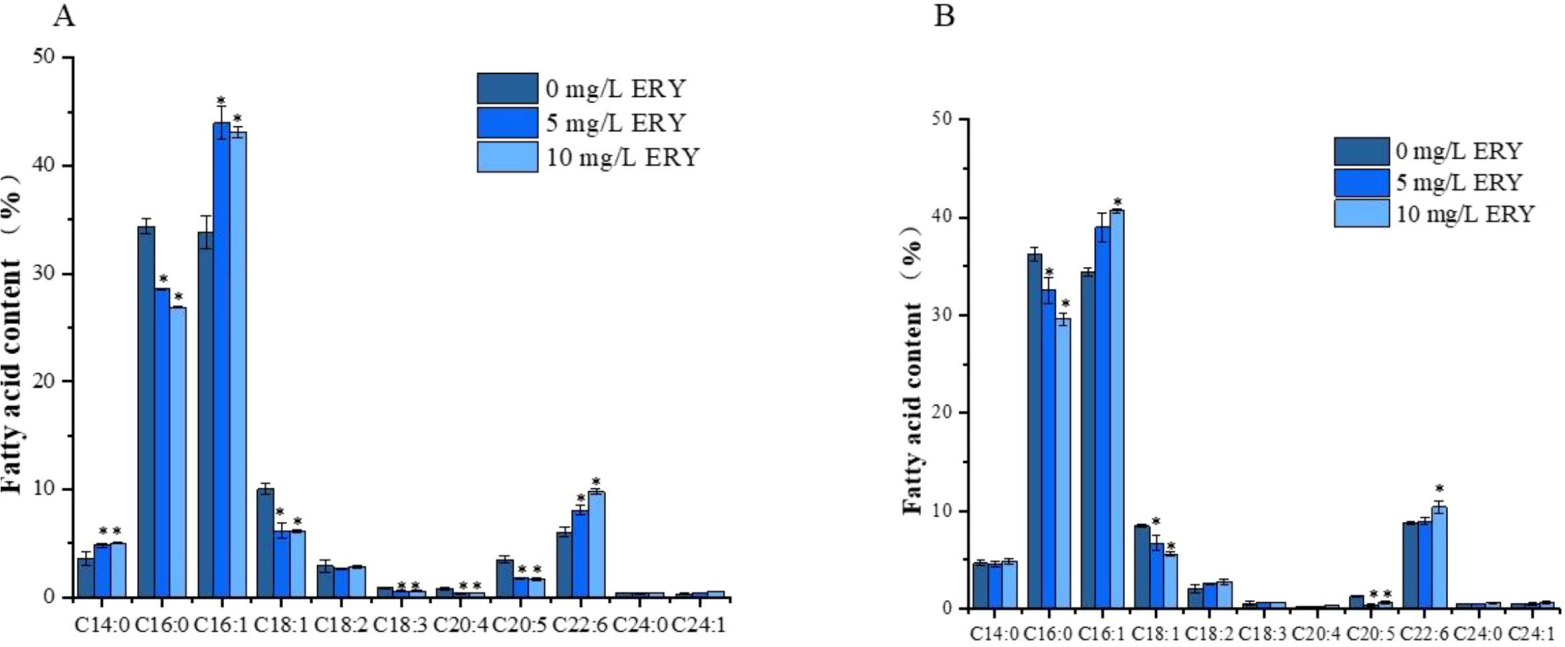
Fatty acid composition and abundance (percentage of total fatty acids) in *P. tricornutum* following a 6-day exposure to different concentrations of ERY under normal (left) and nitrogen-deficient (right) culture conditions. *P<0.05. Data are presented as mean ± SD (n = 3).

The ERY -induced variations of fatty acid composition in lipid droplets were also investigated under both normal and nitrogen-deficient conditions. According to **Figure 5**, **Supplementary Table S1**, under the normal culture condition, palmitic acid (C16:0), monounsaturated palmitoleic acid (C16:1), monounsaturated oleic acid (C18:1), docosahexaenoic acid (C22:6), myristic acid (C14:0), and eicosapentaenoic acid (C20:5) had the highest abundance. However, under exposure to 5 and 10 mg/L ERY, percentages of unsaturated fatty acids were elevated to 66.25% and 67.63%, respectively. Consistent results were also observed under the nitrogen-deficient condition. With exposure to 10 mg/L ERY under the normal culture condition, we found that C16:0 and C18:1 decreased by 7.49% and 3.92%, while C16:1 and C22:6 increased by 9.32% and 3.74%, respectively, indicating a significant change of fatty acid composition. Similar variations in their content were also shown when the nitrogen-deficient culture condition.

**Figure 6:**
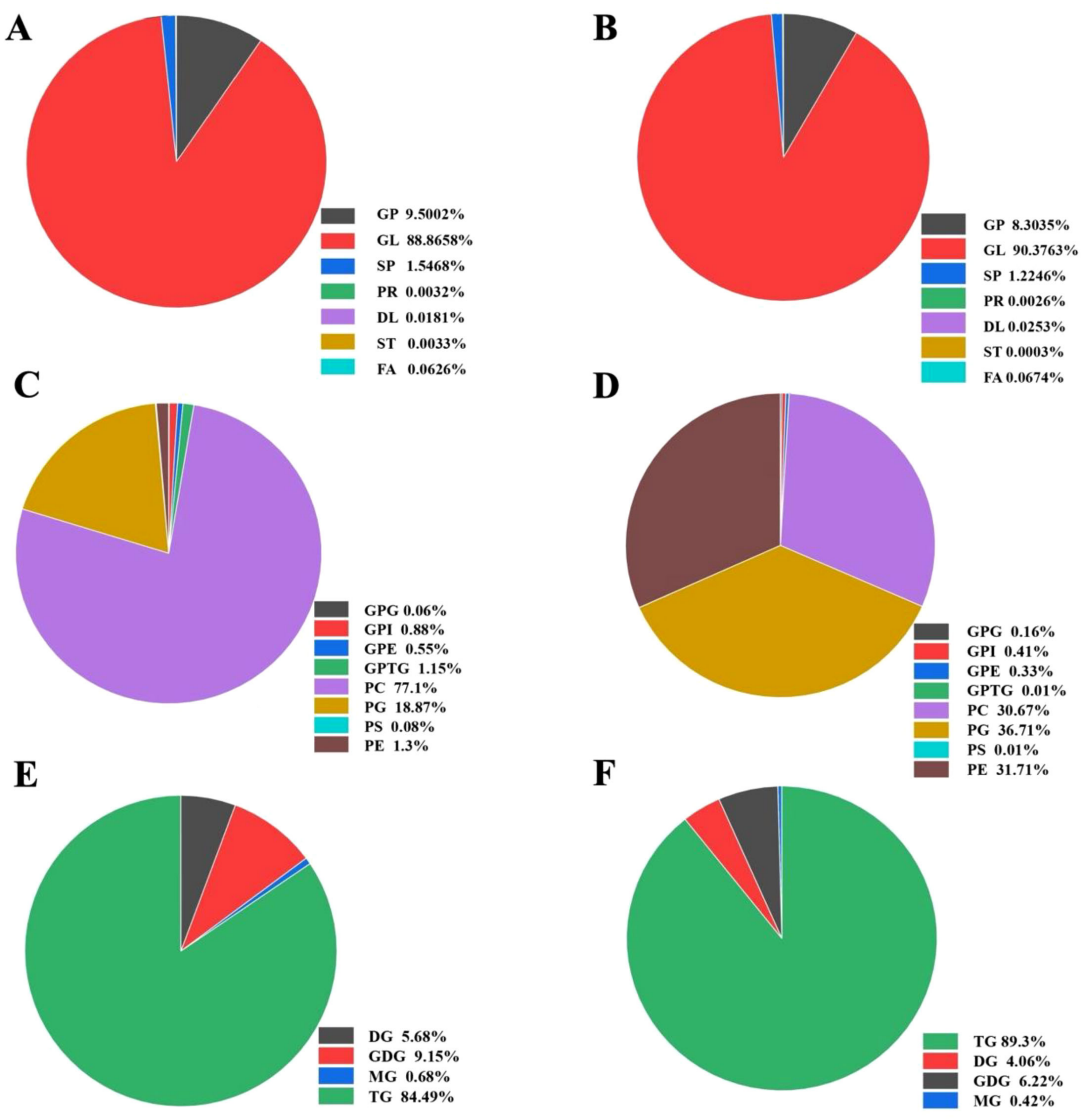
Proportions of different lipid categories in *P. tricornutum* **(A, C, E)** without and **(B, D, F)** with 10mg/L ERY treatment for 6 days. **(A, B)** represent the classification of total lipid metabolites; **(C, D)** represent the glycerophospholipids category; **(E, F)** represent the glycerolipids category. GL, glycerolipids; FA, fatty acyls; GP, glycerophospholipids; SP, sphingolipids; ST, sterol lipids; PR, prenol lipids; DL, derivatized lipids; GPG, glycerophosphoglyceride; GPI, glycerophosphoinositol; GPE, glycerophosphate ethanol; GPTG, glycerophosphatidylglycerol; PC, phosphatidylcholine; PG, phosphatidylglycerol; PS, phosphatidylserine; PE, phosphatidylethanolamine; DG, diglyceride; MG, monoglyceride; TG, triglyceride; GDG, glycolipid diacylglycerol.

The lipidomic profile of *P. tricornutum* significantly changed after treatment with 10 mg/L ERY for 6 days (**Figure 6**). Among the seven classes lipid, GL, GP, and sphingolipids (SP) accounted for the highest proportions. In microalgal cells exposed to 10 mg/L ERY, GL increased by 1.51%, while GP and SP decreased by 1.20% and 0.32%, respectively. At the level of lipid subclasses of GP, 10 mg/L ERY significantly induced the production of PE and PG (increasing by 30.41% and 17.84%, respectively), but significantly inhibited PC production (decreasing by 46.43%). At the level of lipid subclasses of GL, TG increased by 4.81% and GDG decreased by 2.93%. Both PLS-DA and PCA results demonstrated great differences in lipidome between experimental group and control group (**Supplementary Figure S4**). **Supplementary Figure 5** summarizes 80 lipid species that were significantly differentially regulated. The major lipid compounds that were markedly upregulated included 10 TGs, 10 PEs, and 5 PCs. Conversely, those that experienced a marked decline included 6 TGs, 7 PAs, 4 FAs, and 6 GDGs. Notably, unsaturated fatty acids exhibited the greatest variations, which was consistent with the HPLC analysis of lipid droplets extracted from *P. tricornutum*.

### 3.6 Transcription status in response to ERY exposure

**Figure 7:**
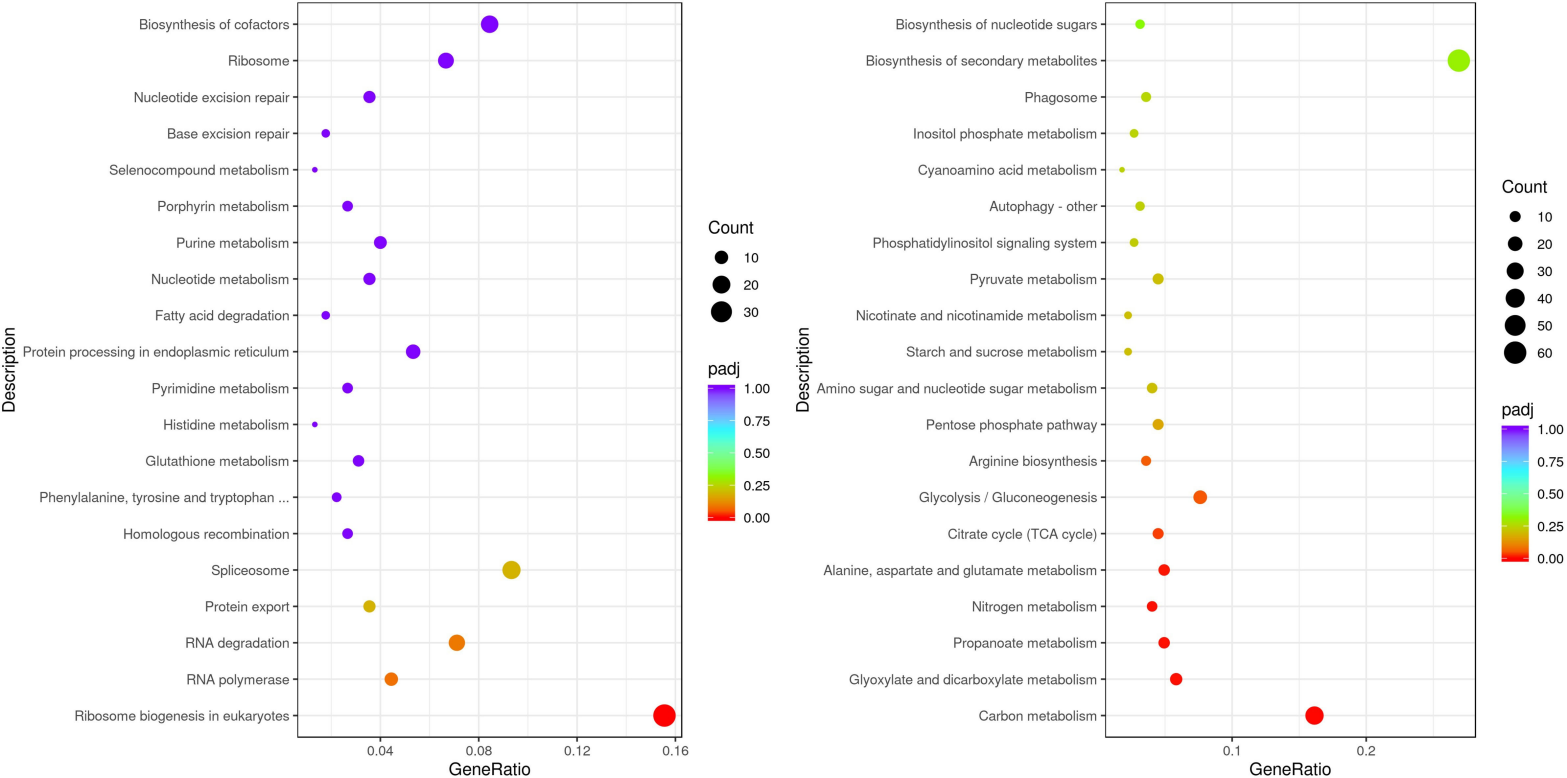
Significantly enriched KEGG pathways of DEGs in *P. tricornutum* exposed to 10 mg/L ERY for 6 d. (left, downregulated genes; right, upregulated genes).

Transcriptome analysis also refers to the transition of metabolism exposed to ERY. A total of 3,540 DEGs, comprising 1,845 up-regulated genes and 1,695 down-regulated genes, were detected (**Supplementary Figure S6**). As shown in **Figure 7**, genes associated with ribosome biogenesis, RNA polymerase, biogenesis of cofactors, protein processing and RNA degradation were significantly down-regulated. These pathways are related to energy generation and cell growth. Pathways including biosynthesis of secondary metabolites, carbon metabolism, glyoxylate and dicarboxylate metabolism, and glycolysis were significantly up-regulated. Pathways directly related with fatty acid synthesis were not detected with differential expression. Notably, fatty acid degradation pathway were downregulated ones under exposure to ERY, which may explain the enhance lipid accumulation.

## 4 Discussion

Previous research demonstrates microalgal susceptibility to ERY varies greatly between 0.03 and 33 mg/L (Lin and Tsai, 2009; Sendra et al., 2018). Marine diatoms exhibit high sensitivity, with an EC_50_ near 0.1 mg/L (Xiong et al., 2020). This is verified by *C. gracili*s and *T. weissflogii*, which are highly sensitive to ERY exceeding 0.5 and 0.75 mg/L (Campa-Córdova et al., 2006; Mo et al., 2023a). Here, high ERY concentrations clearly reduced *P. tricornutum* cell density within 96 h, sustaining this influence until culture completion. Because the ERY concentration exceeded both the growth inhibition EC_50_ (1.31 mg/L) and the effective quantum yield inhibition EC_50_ (8.26 mg/L), no hormesis effect occurred (Xiong et al., 2020). In this study, environmental ERY at 10 mg/L reduced cell density by 45.58% within 96 h, suggesting ERY-induced stress surpasses tolerance limits. However, the growth inhibition was measured by detecting OD_680_. Under the ERY stress, fucoxanthin-chlorophyll-protein in *P. tricornutum* cells undergo drastic remodeling. Thus, OD_680_ can indicate the growth tendency, but not the accurate biomass.

ERY stress significantly increases ROS in microalgae, leading to the oxidation of pigments, membrane lipids, and nucleic acids (Sendra et al., 2018; Du et al., 2020; Pires et al., 2021; Wang et al., 2021; Mo et al., 2023b). In *P. tricornutum*, as previously observed in *M. flos-aquae*, high ERY concentrations triggered a persistent rise in ROS and MDA over 10 d (Wan et al., 2015). Although SOD and CAT were elevated, this continuous accumulation indicates a severe antioxidant system imbalance (Feijão et al., 2020). Consequently, excessive ROS caused structurally impairing ribosomes, mitochondria, and respiratory chains to inhibit growth (Yang et al., 2013; MaChado and Soares, 2019). Concurrently, ERY caused a dose-dependent decline in essential photosynthetic pigments (Kuczynska et al., 2015; Wang et al., 2021). This depletion stems from ROS-triggered degradation into pheophytin a, coupled with ERY interfering with chlorophyll biosynthesis and thylakoid localization (Cavalier-Smith, 2018; Liu et al., 2011; Feijão et al., 2020). Ultimately, massive ROS overwhelms carotenoid antioxidant capacities, causing irreversible oxidative damage, altered membrane permeability, and eventual cell death (Jahns and Holzwarth, 2012; Feijão et al., 2020).

Variations in *P. tricornutum* growth due to stressors are frequently coupled with altered fatty acid composition (Cabrita et al., 2018; Feijão et al., 2018; Duarte et al., 2019; Wang et al., 2021). Our findings indicate detrimental ERY conditions increased lipid synthesis while decreasing biomass. Increased lipid accumulation generally acts as an adaptive self-protection mechanism against adverse stimuli (Xin et al., 2010; Brindhadevi et al., 2021). ERY enhances intracellular lipids in *S. obliquus*, and biodegradation remains the primary ERY degradation factor in wastewater (Du et al., 2020; Wang et al., 2021; Grada et al., 2022; Demir-Yilmaz et al., 2023). Lipophilic ERY may penetrate the cell wall via passive diffusion or transport. Interestingly, the contents of PUFAs in *T. obliquus* exposed to ERY were significantly increased, while the contents of total saturated fatty acids and monounsaturated fatty acids were significantly decreased. Further investigation is required to confirm whether increased PUFAs facilitate the biodegradation (Du et al., 2020). However, the alleviated growth inhibition caused by ERY under nitrogen-deficient culture was not related to the ERY removal.

Diverse fatty acids in membrane and storage lipids govern membrane fluidity and signaling, while increased unsaturated fatty acids reflect a pronounced stress response (Feijão et al., 2018). In *P. tricornutum*, common fatty acids and long chain fatty acids are synthesized in the chloroplast and endoplasmic reticulum, respectively (Kern and Guskov, 2011; Mizusawa and Wada, 2012; Dolch and Maréchal, 2015). Meanwhile, GP classes altered significantly: PE and PG increased, while PC decreased. Stress-induced TG redistribution into lipid droplets is an adaptive response, as TG destabilizes membranes and accumulates with carotenoids (Lupette et al., 2019; Avidan et al., 2021). GDG species are signature photosynthetic lipids forming plastid thylakoids, while PC and PE are major mitochondrial and ER membrane constituents (Tomečková et al., 2020; Shishova and Yemelyanov, 2021). Under ERY exposure, GDG down-regulation and GP redistribution remodeled key organelle membranes, causing altered metabolism.

Binding of ERY to the 50S ribosomal subunit caused severe hamper to pathways about ribosome biogenesis, RNA and protein processing, as shown in **Figure 7** (Nie et al., 2013). Interestingly, fatty acid synthesis at the transcriptome level were not pronounced, with reduced fatty acid degradation pathway instead causing enhanced lipid accumulation. From another perspective, the up-regulated carbon metabolism, glycolysis, and pentose phosphate pathway provide the precursors and reducing power for fatty acid synthesis (Levitan et al., 2015). Bezafibrate exposure reduce O_2_ generation, CO_2_ fixation and fatty acid content, stimulating mixotrophic metabolism rather than directly altering lipid metabolism (Duarte et al., 2019). ERY elevated ROS level, induced acetyl-CoA carboxylase activity, and decreased ribulose-1,5-biphosphate carboxylase activity, causing increased lipid production in green alga (Wang et al., 2021). In this study, further molecular evidence should be obtained to demonstrate the specific step for enhancing lipid production in *P. tricornutum* with ERY exposure.

## 5 Conclusion

This study demonstrates that high ERY concentrations significantly disrupt marine diatom growth, pigment composition, with enhanced antioxidant system. Moreover, the lipid content was increased, with altered fatty acid composition and lipid kinds. Transcriptomics results showed that the enhanced lipid accumulation was attributed to reduced fatty acid degradation, and increased precursors and reducing power. Further molecular evidence should be obtained to demonstrate the specific metabolic reaction, which disturbed by ERY.

## Data Availability

The data presented in the study are deposited in the NCBI repository, accession number PRJNA1453486.
