# Supplementary material for: Erythromycin induces lipid redistribution and toxicological effect of the marine diatom Phaeodactylum tricornutum
Source: Front Plant Sci. 2026 Apr 20;17:1794615. doi: 10.3389/fpls.2026.1794615 (PMC13136097; doi:10.3389/fpls.2026.1794615)
Supplement: Supplementary file 1 [file DataSheet1.docx]

**
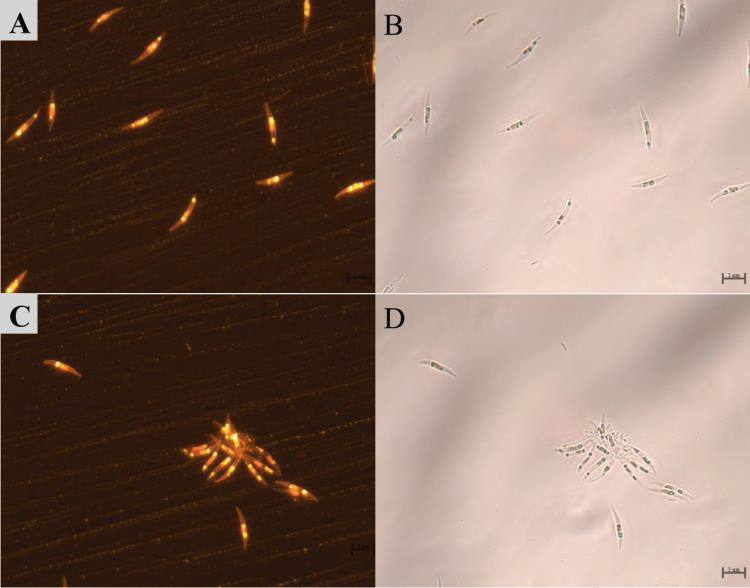
**

**
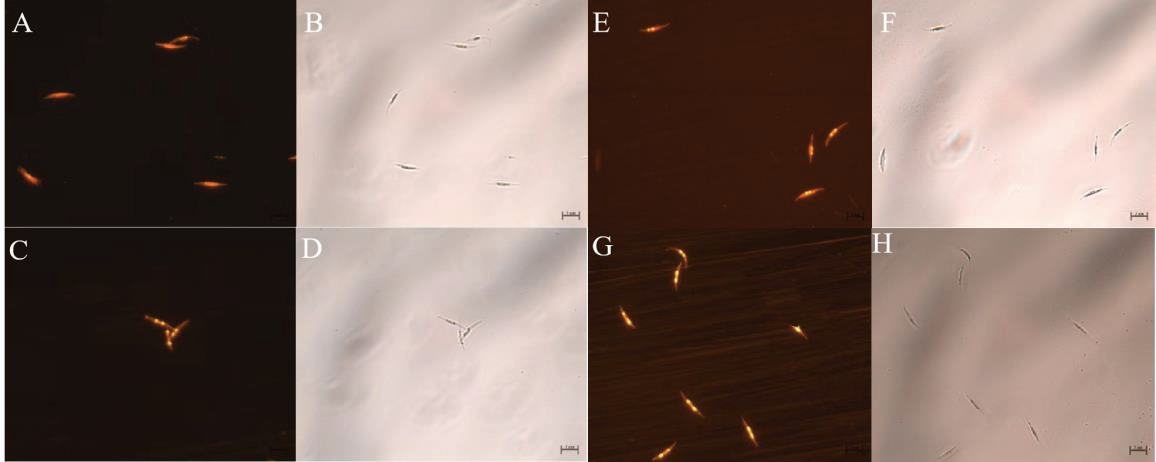
**Fig.S1. Microscopic imaging on Nile red staining of *P. tricornutum* cells in control group (A/B) and 40 mg/L erythromycin treated group (C/D) for 10 days. The scale bar represents 2 μm.

Fig.S2. Microscopic imaging on Nile red staining of *P. tricornutum* cells at 48h on 10 mg/L erythromycin under normal (A/B) and nitrogen-deficient conditions (C/D). The scale bar represents 2 μm.

Fig.S3. Residue rate of ERY in *P. tricornutum* culture medium under normal (left) and nitrogen-deficient conditions (right). Data are presented as mean ± SD (n = 3).

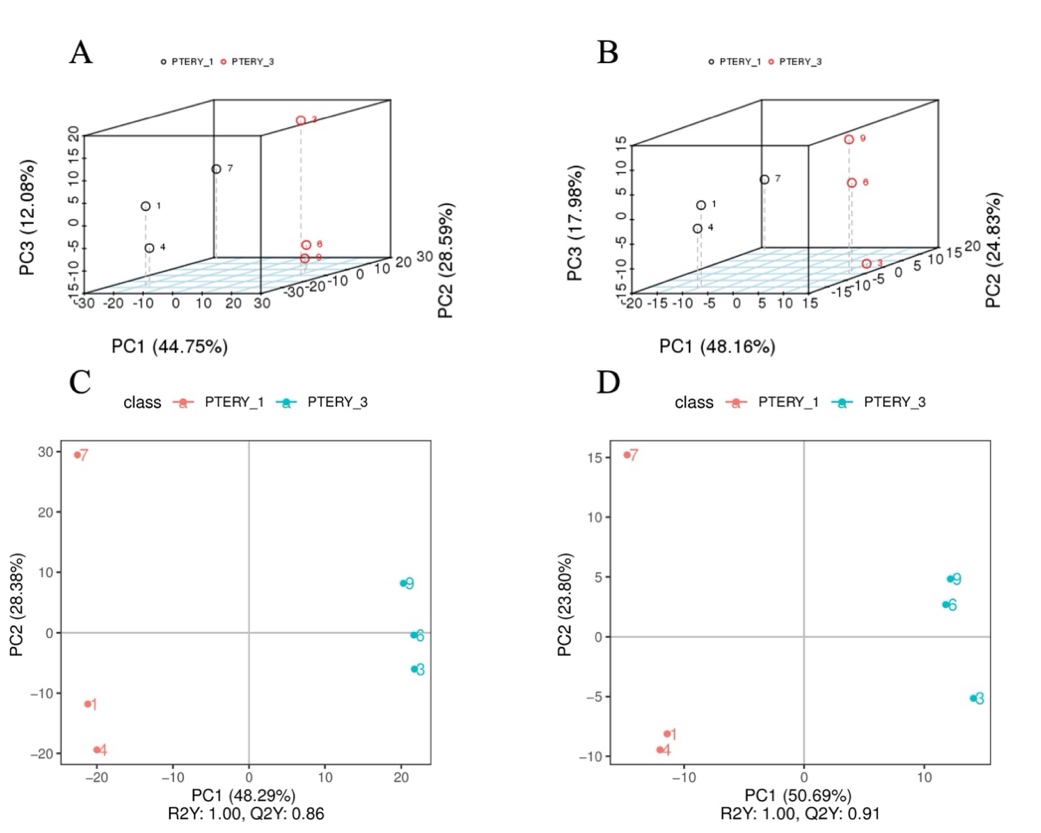


Fig.S4. PCA (A /B) and PLS-DA (C/D) results in positive (A/C) and negative (B/D) ion mode. PTERY1 and PTERY3 represent the control group, and 10 mg/L erythromycin treated group.


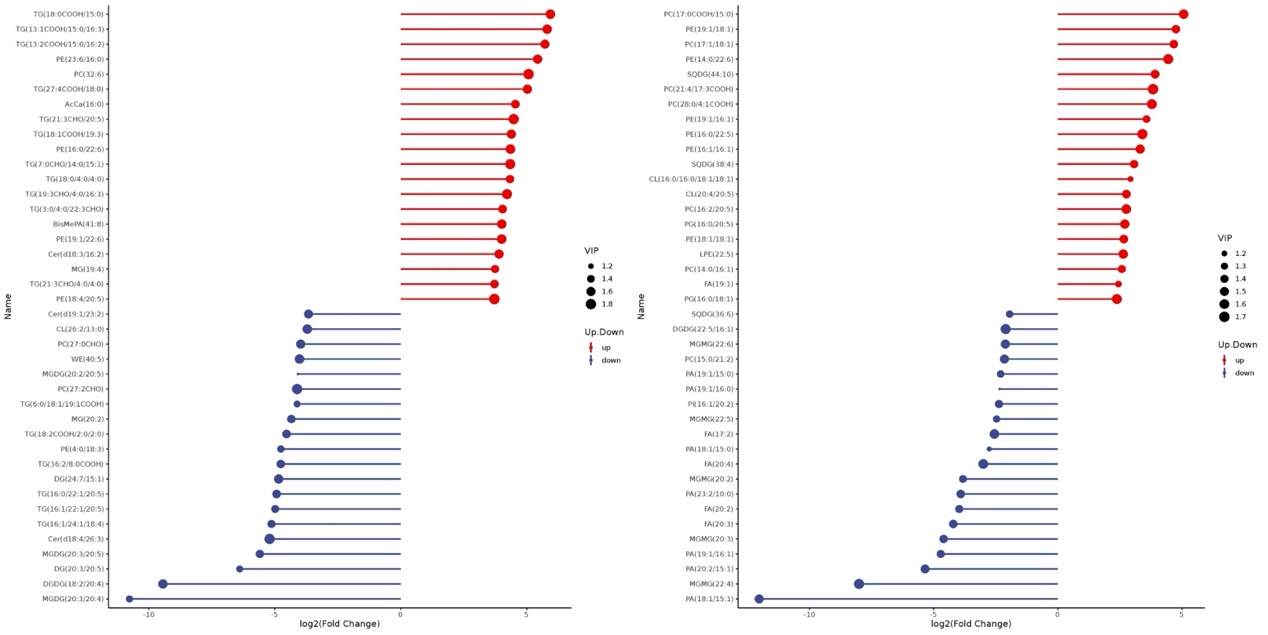
 Fig.S5. General distribution of differential lipids detected in *P. tricornutum* under 10 mg/L ERY treated group for 6 days (left: positive ion mode, right: negative ion mode). Each dot represents one differential lipid. Red indicates significantly increased lipid subclasses, and blue indicates significantly decreased lipid subclasses. TG: triglyceride, PE: phosphatidylethanolamine, PC: phosphatidylglycerol, Cer: ceramide, MG: monoglyceride, MGDG: monogalactosyl diacylglycerol, SQDG: sulfoquinovosyl diacylglycerol, DGDG: digalactosyl monoacylglycerol, FA: fatty acid, CL: cardiolipin, LPE: lysophosphatidylethanolamine, MGDG: monogalactosyl diacylglycerol, PA: palmitic acid, AcCa: acetylated capric acid, Bis-MePA: bis-methyl palmitic acid‌.

**
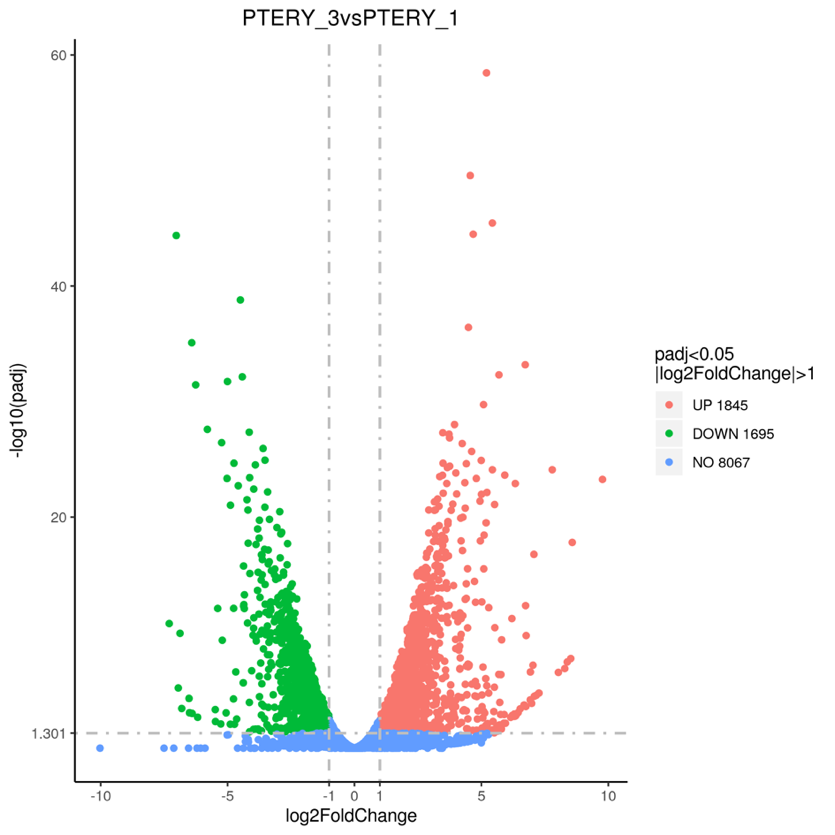
**

Fig.S6. Volcano plot of differentially expressed genes in *P. tricornutum* between the 10 mg/L erythromycin-treated group and the control group for 6 days. Horizontal dashed line indicates the p < 0.05, and vertical dashed line represents the log_2_FC = −1 (left) and log_2_FC = 1 (right) threshold. Genes that are significantly upregulated and downregulated are shown in red and green, respectively, and non-significantly differentially expressed genes are displayed in blue.

Table S1 Fatty acid composition and abundance in *P. tricornutum* following a 6-day exposure to different concentrations of erythromycin

| Fatty acid  (%) | Erythromycin concentration (mg/L) | | | | | |
| --- | --- | --- | --- | --- | --- | --- |
|  | control | 5 | 10 | 20 | 30 | 40 |
| C14:0 | 3.60±0.68 | 4.82±0.18 | 5.04±0.07 | 5.79±0.77 | 5.26±0.64 | 4.31±0.52 |
| C16:0 | 34.38±1.68 | 28.57±0.06 | 26.89±0.06 | 24.08±0.29 | 23.52±2.03 | 23.89±3.66 |
| C16:1 | 33.82±1.49 | 43.97±1.55 | 43.14±0.51 | 38.93±2.98 | 33.67±3.96 | 24.10±0.84 |
| C18:1 | 10.04±0.54 | 6.14±0.70 | 6.12±0.13 | 7.60±1.61 | 8.71±1.03 | 12.55±0.91 |
| C18:2 | 2.92±0.55 | 2.64±0.03 | 2.83±0.14 | 2.90±0.42 | 3.55±0.51 | 4.39±0.90 |
| C18:3 | 0.88±0.03 | 0.60±0.02 | 0.59±0.04 | 0.38±0.05 | 0.33±0.05 | 0.31±0.08 |
| C20:4 | 0.81±0.10 | 0.35±0.04 | 0.43±0.03 | 0.49±0.05 | 0.59±0.07 | 0.50±0.09 |
| C20:5 | 3.52±0.30 | 1.76±0.08 | 1.63±0.13 | 0.99±0.08 | 0.72±0.39 | 0.64±0.22 |
| C22:6 | 6.04±0.41 | 8.08±0.45 | 9.78±0.28 | 12.44±1.08 | 14.72±1.93 | 13.45±0.63 |
| C24:0 | 0.41±0.03 | 0.36±0.08 | 0.44±0.01 | 0.73±0.20 | 1.08±0.08 | 1.60±0.48 |
| C24:1 | 0.31±0.05 | 0.41±0.04 | 0.56±0.03 | 1.24±0.51 | 1.97±0.40 | 2.78±0.40 |
